# Real-Time Radar-Based Hand Motion Recognition on FPGA Using a Hybrid Deep Learning Model

**DOI:** 10.3390/s26010172

**Published:** 2025-12-26

**Authors:** Taher S. Ahmed, Ahmed F. Mahmoud, Magdy Elbahnasawy, Peter F. Driessen, Ahmed Youssef

**Affiliations:** 1 Radar Department, Military Technical Collage, Cairo 11588, Egypt; taher.sayed@ieee.org (T.S.A.); ahmed.fathi@ieee.org (A.F.M.); 2 Technical Research Center (TRC), Cairo 11727, Egypt; m.elbahnasawy@ieee.org; 3 Department of Electrical and Computer Engineering, University of Victoria, Victoria, BC V8P 5C2, Canada; peterd@uvic.ca

**Keywords:** hand motion recognition, radar sensing, clutter reduction, image binarization, feature extraction, hybrid DL model, vitis AI deployment, FPGA acceleration, deep learning processor unit

## Abstract

Radar-based hand motion recognition (HMR) presents several challenges, including sensor interference, clutter, and the limitations of small datasets, which collectively hinder the performance and real-time deployment of deep learning (DL) models. To address these issues, this paper introduces a novel real-time HMR framework that integrates advanced signal pre-processing, a hybrid convolutional neural network–support vector machine (CNN–SVM) architecture, and efficient hardware deployment. The pre-processing pipeline applies filtration, squared absolute value computation, and normalization to enhance radar data quality. To improve the robustness of DL models against noise and clutter, time-series radar signals are transformed into binarized images, providing a compact and discriminative representation for learning. A hybrid CNN-SVM model is then utilized for hand motion classification. The proposed model achieves a high classification accuracy of 98.91%, validating the quality of the extracted features and the efficiency of the proposed design. Additionally, it reduces the number of model parameters by approximately 66% relative to the most accurate recurrent baseline (CNN–GRU–SVM) and by up to 86% relative to CNN–BiLSTM–SVM, while achieving the highest SVM test accuracy of 92.79% across all CNN–RNN variants that use the same binarized radar images. For deployment, the model is quantized and implemented on two System-on-Chip (SoC) FPGA platforms—the Xilinx Zynq ZCU102 Evaluation Kit and the Xilinx Kria KR260 Robotics Starter Kit—using the Vitis AI toolchain. The system achieves end-to-end accuracies of 96.13% (ZCU102) and 95.42% (KR260). On the ZCU102, the system achieved a 70% reduction in execution time and a 74% improvement in throughput compared to the PC-based implementation. On the KR260, it achieved a 52% reduction in execution time and a 10% improvement in throughput relative to the same PC baseline. Both implementations exhibited minimal accuracy degradation relative to a PC-based setup—approximately 1% on ZCU102 and 2% on KR260. These results confirm the framework’s suitability for real-time, accurate, and resource-efficient radar-based hand motion recognition across diverse embedded environments.

## 1. Introduction

Artificial intelligence (AI) has become an integral component of modern life, with significant applications in healthcare monitoring, human–computer interaction, and assistive technologies. Hand motion recognition (HMR) has many practical applications, such as contactless control in smart homes and gesture-triggered alerts in elder care, and it is particularly beneficial for individuals who are deaf or hard of hearing, offering enhanced communication capabilities through the use of wearable and optical sensors [1]. However, traditional HMR systems based on wearable sensors (e.g., gloves and wristbands) or optical sensors (e.g., RGB cameras and depth cameras) face several limitations. Wearable sensors often require cumbersome connections to computers, while optical sensors are sensitive to lighting conditions and raise privacy concerns [2,3]. Radar sensor-based HMR systems have emerged as a promising alternative, offering robustness to environmental variations and preserving user privacy [4].

Table 1 summarizes the key features and limitations of camera-based, Leap Motion, wearable, capacitive proximity, and radar-based hand motion recognition systems. The qualitative categories “High”, “Medium”, and “Low” are assigned based on widely reported operational characteristics in the literature. Specifically, lighting sensitivity reflects dependence on ambient illumination, privacy concerns relate to the capture of visual or identifiable user data, real-time processing indicates typical end-to-end system latency, ease of use accounts for user interaction and setup complexity, and cost follows commonly reported commercial pricing and hardware requirements for each sensing modality.

Radar systems demonstrate superior performance in terms of robustness to lighting conditions, privacy preservation, and real-time processing capabilities [7]. In recent years, radar-based gesture recognition has gained substantial attention due to its ability to capture micro-Doppler signatures, range–time patterns, and fine motion dynamics that optical sensors often fail to extract [8,9]. Deep learning (DL) approaches—including convolutional neural networks (CNNs), CNN–long short-term memory (LSTM) hybrids, and spectrogram-based models—have demonstrated strong performance for radar HMR [10,11]. However, these architectures typically require large datasets, high computational resources, or complex temporal modeling, which limits their suitability for low-power embedded real-time deployment. Furthermore, most existing radar HMR studies report offline evaluations on PC-based platforms rather than on dedicated FPGA hardware, leaving a gap in practical, energy-efficient implementations.

Despite the advantages of radar-based HMR, it faces significant challenges related to data quality and quantity [12]. The limited size of radar-based hand motion datasets and the presence of interference and clutter in radar signals hinder the development of accurate and efficient DL models [13]. To address these limitations and the gap in embedded real-time processing, this paper proposes a novel lightweight DL hybrid framework tailored for radar data. The model is specifically designed to eliminate temporal layers such as LSTMs, reduce computational complexity, and enable practical deployment on FPGA hardware while maintaining competitive accuracy. The main contributions of this work are summarized as follows:

1. A novel radar signal pre-processing pipeline is proposed, combining clutter filtering, energy enhancement, normalization, and image binarization to generate compact and noise-resilient representations that enable high-accuracy hand motion recognition in cluttered radar environments.
2. A lightweight CNN-based feature extraction architecture is proposed that eliminates recurrent layers (e.g., LSTM), reducing the number of trainable parameters by approximately 75% compared to CNN–LSTM–SVM baselines, while maintaining high recognition accuracy.
3. A hybrid CNN–SVM classification framework is proposed, combining deep CNN feature extraction with an optimized multi-class SVM to enhance class separability and decision robustness, achieving a peak classification accuracy of 98.91% on a benchmark UWB radar hand motion dataset.
4. Extensive experimental evaluation and ablation studies are conducted to quantify the impact of each system component. Compared with state-of-the-art radar-based HMR methods, the proposed framework achieves competitive or superior accuracy while reducing training and prediction times by 29.6% and 32.6%, respectively.
5. Real-time deployment of the proposed framework is demonstrated on two heterogeneous SoC-FPGA platforms—Zynq ZCU102 (AMD, San Jose, CA, USA) and Kria KR260 (AMD, San Jose, CA, USA)—using the Vitis AI toolchain, achieving end-to-end accuracies of 96.13% and 95.42%, respectively, along with up to 70% reduction in execution time and up to 74% improvement in throughput compared to PC-based implementations.

The sections of this paper are organized as follows: Section 2 surveys recent research on radar target recognition and discusses its application to hand motion identification. Section 3 describes the methodology of the proposed model architecture. Section 4 details the experimental work, including dataset acquisition and pre-processing. Section 5 presents and discusses the results, while Section 6 concludes the paper and outlines future research directions.

## 2. Literature Review

Radar-based hand motion recognition generally follows a structured processing pipeline consisting of five major stages, each of which plays a critical role in ensuring accurate and reliable gesture classification. It is important to note that most existing radar HMR studies implicitly incorporate all stages of this pipeline—data acquisition, pre-processing, feature extraction, and classification—even if each publication emphasizes only certain components. Accordingly, the following review discusses prior work in a unified manner that reflects how the complete pipeline is typically addressed in the literature. Existing radar HMR studies have employed a variety of methodologies—ranging from traditional feature engineering to deep learning models such as CNNs, CNN–LSTMs, and spectrogram-based networks—with reported accuracies typically between 85% and 97%. However, most works rely on computationally heavy architectures, require large datasets, or perform only offline PC-based evaluation, which restricts their suitability for real-time embedded deployment and motivates the need for lightweight models such as the proposed CNN–SVM framework.

In order to achieve high accuracy in training and classification results, real-world data collection for radar HMR necessitates consideration of the operational scenario, which includes a wide range of environments. Data must be captured under different conditions to reflect gesture variability [14]. The utilization of multiple radars may generate clutter and interference, requiring synchronization, tagging, calibrated sensor placement, and controlled recording procedures [15].

Data pre-processing plays a crucial role in cleaning and preparing radar measurements without compromising their structure or information content [16]. Noise reduction is commonly achieved through background subtraction, loop-back filtering, and other clutter mitigation approaches. Prior studies have analyzed UWB radar processing techniques, including anomaly detection, background subtraction [17], loop-back filtering [18], and moving-target indication filters [19,20], all of which aim to enhance target detectability by suppressing static clutter and self-interference. These pre-processing stages convert raw radar data into forms that are more suitable for feature extraction and classification.

The selection of an appropriate feature extraction method depends heavily on application constraints such as computational complexity, robustness, and required precision. Time-domain features (e.g., peak amplitude and ToA) are lightweight but less expressive, whereas frequency-domain and time–frequency representations (e.g., spectrograms and CVD) provide richer motion signatures at higher computational cost [21,22,23]. Machine learning-based feature extraction using CNNs and RNNs [24] enables end-to-end learning of discriminative patterns but typically introduces large model sizes and long training times. In this work, lightweight CNN-based extraction is adopted to balance accuracy and efficiency, followed by an SVM classifier to improve decision boundaries while maintaining compatibility with FPGA deployment [25].

Classification of radar-based gestures has been explored using logistic regression, decision trees, random forests, and SVMs [26,27,28]. Although hybrid CNN–LSTM–SVM frameworks have achieved strong performance, their reliance on recurrent layers leads to high computational and memory burdens [29,30]. These limitations limit real-time embedded applicability. In contrast, the proposed CNN–SVM framework maintains high accuracy while reducing computational cost, making it well-suited for deployment on low-power SoC FPGA hardware [31,32,33].

Despite the progress achieved in radar-based HMR, existing studies share several noteworthy limitations. Many deep learning approaches rely on large annotated datasets that are difficult to obtain for radar applications, and their performance degrades when training data are limited. Architectures such as CNN–LSTM, attention mechanisms, and multi-branch spectrogram networks introduce high computational cost and memory overhead, making them unsuitable for real-time embedded inference. Furthermore, several reported methods are sensitive to clutter, interference, and variations in gesture execution, particularly when multiple radars operate simultaneously. Importantly, the majority of prior works evaluate their models only offline on CPU/GPU platforms, with little emphasis on power-efficient FPGA deployment. These limitations highlight a clear need for lightweight, clutter-resilient, and hardware-efficient radar HMR models, which this work aims to address.

Beyond radar-based HMR, robustness and resource-efficient recognition have also been explored in other security-critical sensing domains. For example, Kwon et al. developed AudioGuard, a speech recognition system resilient to optimized adversarial audio attacks, demonstrating how malicious perturbations can affect predictions and how defenses improve reliability [34]. Similarly, Friend-Guard introduces targeted adversarial noise for EEG-based brain–computer interface spellers to protect privacy [35], while CloudNet presents a LiDAR-based face anti-spoofing model robust to lighting changes [36]. Despite relying on different sensing technologies, these works similarly aim to enhance noise robustness and support efficient deployment on limited-resource hardware.

Several recent studies have explored deep learning architectures for radar-based hand motion recognition and serve as important benchmarks for performance comparison. CNN-based ensemble trees (CNN-ETs) were investigated in [37] to enhance robustness through a combination of handcrafted and learned features, although with relatively limited validation accuracy. Hybrid CNN–LSTM–SVM architectures were proposed in [28] to capture temporal dependencies in radar signals, achieving high recognition performance at the expense of increased computational complexity. End-to-end CNN classifiers with Softmax outputs were reported in [38], demonstrating strong training accuracy but reduced generalization capability. More advanced approaches, such as neural architecture search-based models (GPNAS) [29] and attention-enhanced CNN–LSTM frameworks [39], achieved high validation accuracy; however, they rely on deep, resource-intensive architectures. While these methods demonstrate strong recognition performance, their reliance on recurrent or attention mechanisms and high computational demand limits their suitability for low-power and cost-effective FPGA deployment, motivating the lightweight CNN–SVM framework proposed in this work.

## 3. Methodology

**Figure 1:**
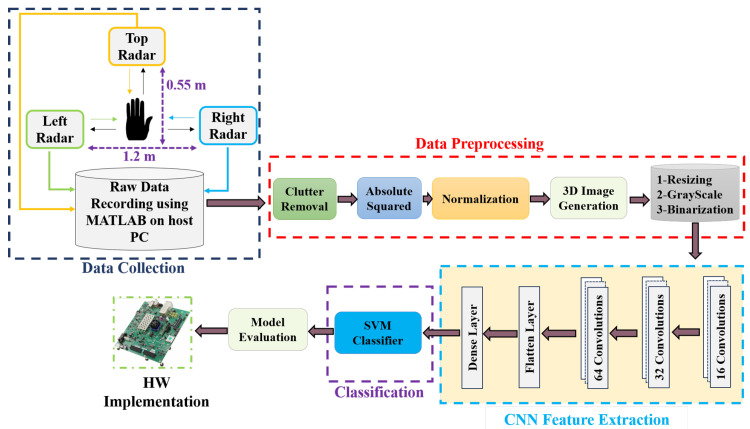
Procedure of HGR phases commencing with the collection of a dataset via UWB radar sensors, followed by dataset preparation according to the proposed methodology, then feature extraction utilizing a hybrid model comprising three concatenated CNNs and an SVM classifier, then proceeding to model evaluation and deployment on the ZCU102 Evaluation Kit (AMD, San Jose, CA, USA).

The methodology diagram shown in Figure 1 illustrates the proposed approach for the acquisition, processing, and classification of radar HMR. The dataset in [40] was collected by utilizing 3 radars (top, left, and right), positioned in three different locations with a distance around 1.2 m between (Left–Right) radars and a distance of 0.55 m between the Top and the center of the horizontal line between other radars.

Radar sensor measurements are captured using MATLAB R2022b on a host personal computer. The subsequent phase, data pre-processing, encompasses eliminating clutter and interference, achieving accurate normalization and generating binarized images to prepare the data for feature extraction and classification.

Feature extraction is performed using three CNNs throughout the third stage. Next, the acquired features are ultimately classified and categorized to the corresponding class. This thorough methodology guarantees that the radar data are carefully processed and examined to produce precise classification outcomes. Finally, the model, developed for classification, is uploaded to an FPGA-based processor for real-time HMR. Unlike the CNN-LSTM-SVM baseline [28], our proposed framework introduces three key differences. First, it eliminates the LSTM layers, which significantly reduces the model size (by more than 70%) and lowers training and inference latency. This simplification is especially important since FPGAs lack native support for recurrent layers, making CNN-only architectures more hardware-friendly. Second, the CNN-SVM design maintains competitive accuracy while requiring fewer parameters, resulting in faster convergence and reduced power consumption compared to the CNN-LSTM-SVM model. Third, while employed a multi-radar configuration, our work evaluates a single-radar scenario to test robustness under constrained sensing conditions and to minimize hardware cost for practical deployment. Moreover, the proposed model does not require heavy data pre-processing or manual feature engineering, which eliminates dependence on expert knowledge and enables streamlined end-to-end learning. The details of these major steps of the proposed approach are described in the following subsections.

### 3.1. Data Collection

**Figure 2:**
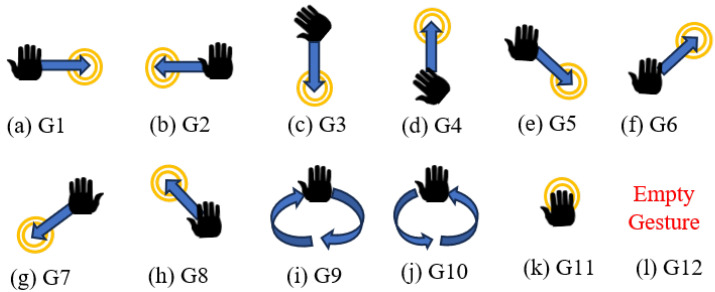
Hand motion vocabulary used to evaluate the classifier.

In this work, to compare results, the UWB-Gestures dataset by authors in [40] is used. This dataset is widely used in radar-based motion recognition research and provides a diverse set of hand motions captured. The dataset was collected as follows. Three XeThru X4 UWB-impulse radars (IR) (Novelda, Oslo, Norway) are utilized and positioned in three different locations (left-top-right) with distances as shown in Figure 1. Eight persons participated in data collection with 12 predetermined hand motions for each one as shown in Figure 2. Motions are coded as listed in Table 2.

In our work using this dataset, the left radar is selected as the main radar for collecting the hand motions, while the other (top and right) radars are considered as sources of clutter and interference. This decision allowed us to evaluate the robustness and effectiveness of our clutter removal and pre-processing pipeline under a more challenging single-sensor scenario. It is important to note that relying on a single radar may inherently limit performance compared to the multi-radar configuration adopted in [41], as fewer sensing perspectives reduce spatial diversity and the ability to suppress ambiguities. Nevertheless, this constraint was deliberately introduced to demonstrate that the proposed framework can still achieve high classification accuracy under resource-constrained conditions. Such validation highlights the practicality of deploying radar-based HMR systems in cost-sensitive or embedded applications where multi-radar setups are not feasible.

### 3.2. Data Pre-Processing

Due to the simultaneous operation and radiation of many radar sensors, initially, a loop-back filter is employed to reduce clutter levels according to Equations (1) and (2).

(1) $$C_{n}\left[k\right]=\alpha \;C_{n}\left[k-1\right]+\left(1-\alpha \right)\;x_{n}\left[k\right]$$

(2) $$y_{n}\left[k\right]=x_{n}\left[k\right]-C_{n}\left[k\right]$$

where $C_{n}$ represents the clutter term, which is extracted using the previously estimated clutter and the current received radar signal x[n], and the alpha (α) term represents the weighting factor that controls the learning rate of the filter. The estimated clutter $C_{n}$ is then subtracted from the received radar signal $x_{n}\left[k\right]$ to obtain the clutter-free output $y_{n}\left[k\right]$. The loop-back filter in Equations (1) and (2) is specifically designed to eliminate reflections originating from static objects such as walls, furniture, and the radar enclosure. These components appear consistently across consecutive pulse repetition intervals (PRIs), allowing the recursive filter to learn a stable clutter estimate $C_{n}\left[k\right]$. Subtracting this estimate removes stationary background energy, leaving only the time-varying components associated with hand motion.

After clutter removal, small residual interference and low-amplitude noise may still be present. To strengthen gesture-specific patterns and improve robustness to outliers, the second pre-processing step applies a squared-absolute transformation. This nonlinear operation amplifies high-energy motion reflections while suppressing low-amplitude clutter and noise, thereby enabling more effective feature extraction. Such energy-based transformations are widely used in radar and signal processing to enhance motion-induced components and attenuate background interference [19]. Consequently, all filtered data samples are squared to increase the level of useful signal relative to background interference, as shown in Equation (3). Each instance, $y_{n}\left[k\right]$, which represents the filtered samples, is squared to obtain $z_{n}\left[k\right]$. Such nonlinear transformations have been reported to improve feature separability and enhance the robustness of learning-based classifiers by highlighting signal variations that would otherwise be less discernible [20,24]. As a result, the proposed model is able to capture more expressive patterns in the radar data.

(3) $$z_{n}\left[k\right]=|x_{n}^{2}\left[k\right]-2x_{n}\left[k\right]c_{n}\left[k\right]+c_{n}^{2}\left[k\right]|$$

The third step of data pre-processing is radar data normalization, which allows for the preservation of numerical stability by minimizing the likelihood of overflow or underflow. Additionally, it reduces the influence of noise by scaling the data to a uniform range, therefore enhancing the visibility of the desirable signal.

The final step in the pre-processing stage is the generation of image representations. First, the normalized radar matrices obtained from the previous step are mapped into RGB images by linearly scaling the signal amplitudes into three channels (R, G, and B), which provides a more discriminative representation of motion energy distribution. These RGB images are then converted into grayscale images to simplify computation without losing essential spatial information. Subsequently, the grayscale images are resized to 75×75 pixels. This resizing step reduces the dimensionality of the input while still preserving the overall motion patterns, thereby lowering computational cost, decreasing training time, and mitigating overfitting risks in lightweight CNN architectures. Finally, the resized images are converted to binary images by applying a fixed threshold, producing compact input samples for the feature extraction phase. This binarization represents a key novelty of the proposed framework, yielding a low-dimensional, clutter-suppressing representation that differs from the spectrogram-based and multi-channel Time–Frequency Representation (TFR) inputs often used in prior radar HMR studies.

### 3.3. Feature Extraction

At this stage, the pre-processed data are analyzed using a sequential CNN model to extract the most discriminative spatial features. The proposed architecture consists of three successive convolutional blocks, each containing a convolutional layer, a max-pooling layer, and a dropout layer. The input to the network is a binarized image of size 75×75, and the three convolutional layers employ filter depths of 16, 32, and 64, with kernel sizes of 4×4, respectively. This progressive design enables hierarchical feature learning as follows: the first convolutional block captures low-level spatial edges and motion gradients, the second extracts mid-level texture and structural cues, and the third encodes high-level abstractions relevant to gesture differentiation. The choice of three convolutional layers follows extensive empirical evaluation, showing that deeper networks offered only marginal accuracy gains while significantly increasing latency and FPGA resource utilization, whereas shallower networks reduced recognition performance. Similar compact architectures have been successfully adopted in radar-based gesture recognition and embedded deep learning applications [28,29,30].

A stride of 2×2 is applied in each convolutional layer to progressively reduce the feature-map dimensions and computational load, while a 2×2 max-pooling operation further condenses spatial information without discarding essential features. Each block includes a dropout layer with a rate of 0.25 to mitigate overfitting and improve generalization, particularly given the moderate dataset size. This layer arrangement was optimized to maintain a balance between expressive feature extraction and efficient hardware implementation. Overall, the sequential CNN structure achieves a compact, hardware-friendly design capable of real-time operation on FPGA platforms, while eliminating the need for recurrent or attention-based layers that would otherwise increase inference latency and power consumption. To justify the selected architectural settings, an architecture sensitivity analysis was conducted by systematically varying the CNN depth, kernel size, and dropout rate, rather than removing system components, in order to assess their impact on classification accuracy and hardware resource utilization. The objective was to determine the most hardware-efficient design that maintains high recognition accuracy. Table 3 summarizes the main results.

The results indicate that the proposed three-layer configuration achieves the best trade-off between accuracy and computational efficiency. Increasing the depth to four convolutional layers yields only a marginal accuracy gain of 1.3% while significantly increasing LUT and BRAM utilization, which negatively affects the maximum achievable clock frequency on the ZCU102 board. Additionally, we tested kernel sizes of 3 × 3 and 5 × 5; the former slightly degraded accuracy, while the latter increased the computational load by approximately 22% without noticeable performance benefit. The selected 4 × 4 kernel provided the best balance across all metrics.

Dropout tuning further revealed that a rate of 0.25 minimizes overfitting while preserving feature expressiveness. Lower dropout values led to overfitting, whereas higher values suppressed critical high-level features. Based on these experiments, the adopted architecture demonstrates a compact and hardware-friendly design that maximizes recognition accuracy while meeting real-time constraints. The empirical findings align with recent embedded CNN design guidelines for radar-based gesture recognition.

### 3.4. Classification

The Support Vector Machine (SVM) is employed as the final classification stage due to its strong generalization capability on compact feature representations. SVM seeks an optimal separating hyperplane by maximizing the margin between classes, and for nonlinearly separable data it relies on kernel functions to implicitly map the input features into a higher-dimensional space where linear discrimination becomes feasible. In this work, the features extracted by the sequential CNN blocks form a well-structured representation that enhances the separability of gesture categories, enabling the SVM to construct robust decision boundaries.

The input for the SVM module for training and testing consists of features generated automatically by the three CNNs using the hand gesture binarized dataset. Iterative training of the multi-class SVM classifier is conducted using the Optuna open source hyperparameter optimization framework, which automates the SVM hyperparameter search to achieve maximum accuracy and minimum losses. The hyperparameters include the penalty coefficient, kernel function, slack variable (degree), and gamma parameter [32]. The kernel parameters determine the shape and structure of the separating hyperplane. SVM hyperparameters are optimized using Bayesian optimization (Optuna library) over a specific number of trials with 5-fold cross-validation on the training set. The search space includes the kernel type k∈{rbf,poly,linear,sigmoid} [33], the regularization parameter C∈[0.1,3] (log scale), the kernel coefficient γ∈{auto,scale}, and the polynomial degree∈{1,2,3}. The objective is the mean validation accuracy across the folds. After 100 iterations of optimizing, the SVM hyperparameters, the penalty coefficient, kernel function, slack variable (degree), and gamma parameter are fixed for the classification process. The combination of CNN-based feature extraction and SVM decision-making results in a hybrid classification pipeline that leverages the representational power of deep learning and the margin-maximization properties of SVM. This structure not only improves recognition accuracy but also enhances generalization across subjects and gesture variations, making it suitable for resource-constrained real-time systems.

### 3.5. Evaluation

The performance of the proposed model is quantified using the accuracy, precision, recall, and F1-score metrics [42]. These metrics are computed by examining the quantities of true positives ($T_{p}$), true negatives ($T_{n}$), false positives ($F_{p}$), and false negatives ($F_{n}$) using the following mathematical formulas.

(4) $$\mathrm{Accuracy}=\frac{T_{p}+T_{n}}{T_{p}+T_{n}+F_{p}+F_{n}}$$

(5) $$\mathrm{Precision}=\frac{T_{p}}{T_{p}+F_{p}}$$

(6) $$\mathrm{Recall}=\frac{T_{p}}{T_{p}+F_{n}}$$

(7) $$\mathrm{F1-score}=\frac{2T_{p}}{2T_{p}+F_{p}+F_{n}}$$

### 3.6. Hardware Implementation

A pre-trained CNN-SVM model is implemented on a DL Processing Unit (DPU) using two FPGA platforms—Zynq ZCU102 and Kria KR260—to evaluate the efficiency of real-time classification. This dual-platform implementation leverages the real-time processing capabilities of FPGA hardware and satisfies the demanding speed and accuracy requirements of hand motion data recognition. The comparative deployment on both platforms demonstrates the framework’s scalability and adaptability across different embedded hardware configurations.

## 4. Experimental  Work

This section illustrates the work executed to implement the proposed methodology for the acquisition, processing, classification, and evaluation of radar-based HMR data. The hardware platforms used in this study include the X4M02 UWB radar sensor (Novelda, Oslo, Norway) for data collection and two FPGA platforms—the Zynq ZCU102 Evaluation Kit (AMD, San Jose, CA, USA) and the Kria KR260 Robotics Starter Kit (AMD, San Jose, CA, USA)—for model deployment and performance evaluation. The proposed workflow consists of the following five main phases:

### 4.1. Phase 1: Data Collection Using UWB Radar Sensor

A system is developed using the X4M02 radar sensor module, which provides a comprehensive radar sensing solution by integrating the XeThru X4 System-on-Chip (SoC) (Novelda, Oslo, Norway), one transmitter (Tx) antenna, one receiver (Rx) antenna, and an onboard microcontroller for application-level signal processing. The radar module enables accurate detection and measurement due to its high-performance specifications. It operates at a center frequency of 8.745 GHz with a bandwidth of 1.5 GHz (−10 dB), a sampling frequency of 23 GHz, and a pulse repetition frequency of 40.5 MHz, while capturing data at 20 frames per second [40].

Each radar frame consists of 1536 samples, corresponding to a spatial resolution $\Delta R_{\mathrm{raw}}$ of approximately 6.51 mm, assuming a maximum observable range of $R_{\max}=10$ m. To concentrate the analysis on the hand-motion region, a Region of Interest (RoI) of 1.2 m is selected. During post-processing, windowing and binning techniques are applied to this RoI, effectively reducing data dimensionality while preserving adequate spatial resolution. Given the raw resolution, the number of resulting range bins, denoted as $N_{\mathrm{bins}}$, is estimated to be around 189. These characteristics make the radar particularly well suited for applications requiring precise motion detection and high spatial accuracy.

For each hand motion, three synchronized radar scattering matrices are acquired, capturing the reflected signal magnitude over time and distance. The resulting 2D matrices consist of 90 slow-time frames (at 20 fps for a duration of 4.5 s) and 189 fast-time samples (covering 1.2 m). These matrices are stored using MATLAB, preserving the raw data to enable subsequent noise reduction and pre-processing. The proposed framework then transforms these raw data into low-dimensional, high-intensity feature representations, which improves classification performance using a computationally efficient deep learning (DL) model.

### 4.2. Phase 2: Dataset Pre-Processing

**Figure 3:**
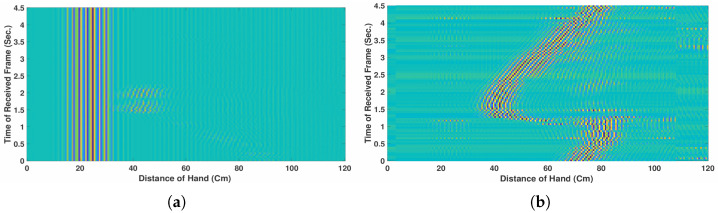
Patterns of G1 recorded motion for a 4.5 s duration. (**a**) Before clutter removal. (**b**) After clutter removal.

Data preparation involves cleaning, organizing, and transforming the raw radar measurements into a suitable format for effective machine learning analysis. For simplicity, only the data from the left radar sensor are used in the current implementation. Figure 3a,b illustrates an example of the G1 (LR-swipe) motion before and after clutter removal. In the presence of clutter, the underlying motion pattern is obscured as shown in Figure 3a, whereas after filtering, the motion trajectory becomes clearly observable as illustrated in Figure 3b.

**Figure 4:**
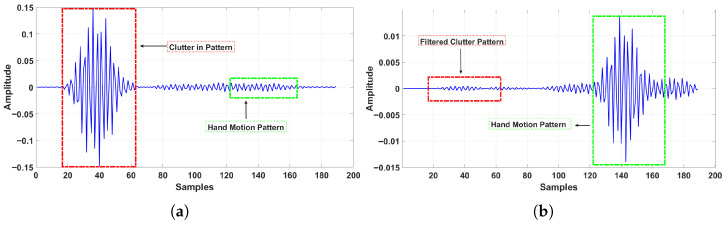
Loop-back filter effect on an LR-swipe sample (#80) recorded at t=4 s by the first participant. (**a**) Raw received PRI signal. (**b**) Clutter-removed PRI signal.

Figure 4 demonstrates the effect of applying the loop-back filter, which mitigates the excessive static reflections present in all received frames. These clutter components originate from stationary objects in the environment—as well as from the radar modules themselves—and from simultaneous radiation at the same operating frequency by the three radars used in the setup. The recursive filter suppresses these slowly varying components by continuously estimating and subtracting them, thereby preserving only the dynamic reflections associated with hand motion.

**Figure 5:**
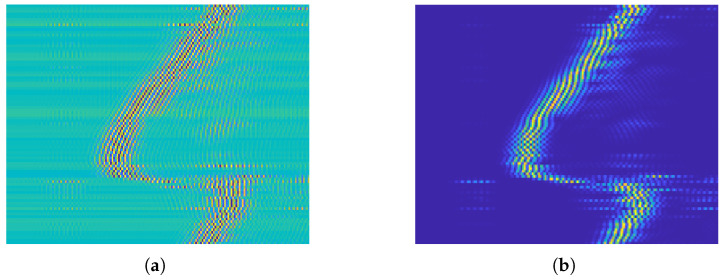
Left-Right swipe sample processed images. (**a**) Filtered (clutter-free) image. (**b**) Filtered, squared absolute, and normalized image.

After clutter suppression, a squared-absolute (energy-based) transformation is applied. Figure 5 compares the filtered (clutter-free) radar image with and without this transformation. The squared-absolute operation enhances high-energy reflections generated by hand movements while attenuating low-amplitude residual noise, resulting in clearer spatial–temporal motion patterns prior to normalization. The motion of the participant’s hand, called “LR-swipe,” in the first half of the matrix corresponds to high-energy reflections collected at closer range bins, which gradually shift rightward as the hand moves further from the radar over time. This rightward shift accurately reflects the temporal progression of the motion/gesture, where reflections begin at near distances and propagate to farther ones as the hand moves away, consistent with the physical definition of G1.

**Figure 6:**
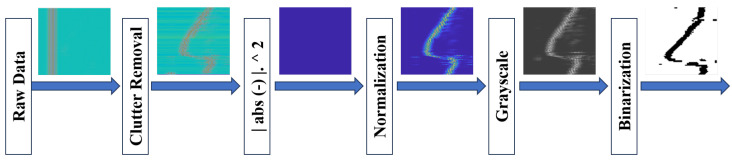
Left-Right swipe sample data preparation step outputs.

Figure 6 shows all pre-processing steps results used for binarized image generation for G1 (left-right swap) as motion sample data. This progression clarifies the transformation of the signal at each stage. In other words, the normalized output is represented as 3D RGB images, which are then converted to grayscale ones followed by a resizing stage to 75×75 to decrease the computational cost and increase model performance. The resized images are then converted to binarized images by applying thresholding before entering the feature extraction phase.

**Figure 7:**
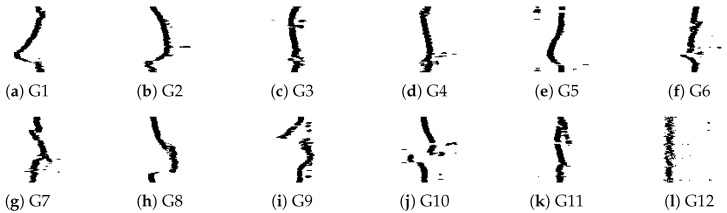
Samples from the binarized Left-UWB-Radar Gestures dataset.

Figure 7 shows a generated binarized sample for all hand gesture classes which are used as the dataset for CNNs. The pre-processing stage produces 700 binarized samples per motion class. The dataset samples are divided so that 80% of the data have been allocated for training the suggested model, 10% have been used for the validation of the trained classifier, and 10% have been assigned for testing the trained classifier.

For highlighting the effect of the full pipeline, a component-wise analysis was conducted to evaluate the impact of the proposed pre-processing chain. Three variants were compared using the same proposed architecture as follows: (i) full pipeline; (ii) without the squared-absolute step (filtering + normalization only); and (iii) normalization only, the results of which will be mentioned in Section 5.

### 4.3. Phase 3: Feature Extraction

The majority of feature extraction approaches in radar-based HMR rely on applying edge-detection filters such as Sobel, Gaussian smoothing, or other gradient-based operators to enhance image features prior to classification. These filtered outputs are typically passed into CNN architectures that include Max-Out layers, and in many cases are followed by long short-term memory (LSTM) networks to model temporal dependencies. In contrast, the proposed feature extraction method adopts a more lightweight and efficient design, eliminating the need for additional filtering or recurrent layers. The key characteristics of the proposed approach are summarized as follows:

- Depending only on the prepared data from phase (2) without using extra filtration layers, such as the Sobel filter, the Guassian filter, and/or other edge-detection filters.
- Using three successive CNNs without a Max-Out layer while many previous works utilize one CNN.
- Removing the LSTM layer, despite the fact that the majority of the literature employs the LSTM layer, and reaching a maximum of 150 layers [28].

**Figure 8:**
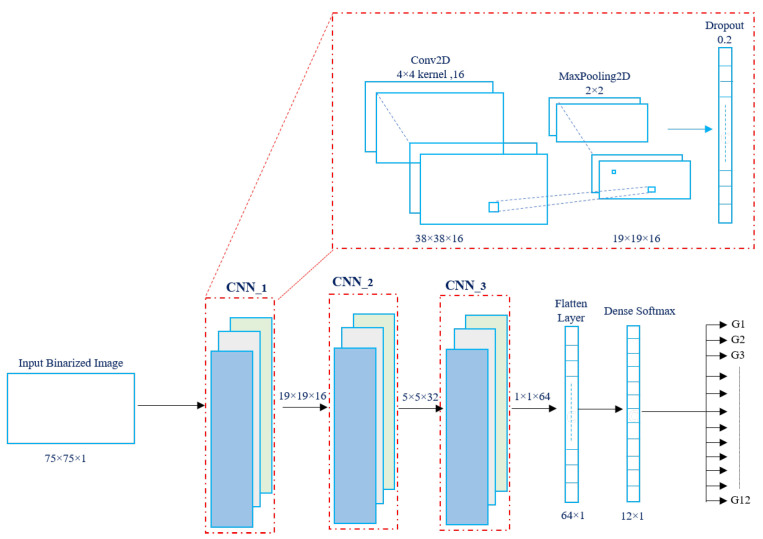
Feature extractor architecture using a hybrid deep learning model composed of three CNNs.

The schematic diagram of the proposed feature extraction model is shown in Figure 8, while the input and output parameters of individual layers are listed in Table 4. From a computational perspective, the proposed CNN-only feature extractor requires approximately $2.97\times 10^{6}$ FLOPs per gesture sample against about $2.57\times 10^{5}$ FLOPs for the LSTM, resulting in a total of approximately $3.23\times 10^{6}$ FLOPs per sample. This corresponds to an 8.6% increase in theoretical operations due to the recurrent component. Despite this modest increase, the CNN-only extractor offers a simpler and more efficient architecture better suited for real-time embedded deployment.

According to Figure 8, the proposed architecture of a CNN is comprised of an input layer, a separable convolutional layer, a max-pooling layer, a dropout layer, a flatten layer, and a softmax layer. The input layer represents the raw input (pixels) in the form of a 2D matrix, while the separable convolutional layer is used to generate the feature map by mixing the input layer with a 2D filter kernel of the necessary size. The max-pooling layer is an additional pooling layer utilized in conjunction with convolutional layers to down-sample feature maps while preserving the essential information. The dropout layer function focuses on reducing the likelihood of overfitting occurring. The flatten layer establishes a connection between the convolutional and fully connected layers of a CNN to transform a multidimensional tensor after several pooling and convolutional procedures into a one-dimensional vector with the retrieved features. The last layer is the softmax layer, which receives the flattened feature vector and produces an output vector with a length equal to the number of target classes (12 classes in this case).

### 4.4. Phase 4: SVM Classifier

The SVM constitutes the final classification stage of the proposed hybrid framework. Its main objective is to determine the optimal separating hyperplane in the high-dimensional feature space generated by the CNN, thereby partitioning the data into the 12 gesture classes. Rather than operating directly on raw radar signals or images, the SVM receives compact and discriminative feature vectors extracted from the CNN in Phase 3.

To ensure robust classification performance, SVM hyperparameters—including the kernel type, regularization parameter *C*, and kernel coefficient γ—were tuned using the Optuna optimization framework over 100 iterations. This automated search identified the optimal configuration that balances margin maximization with generalization capability. During the hyperparameter optimization process, multiple SVM kernel functions—including linear, polynomial, radial basis function (RBF), and sigmoid—were systematically evaluated. The RBF kernel consistently outperformed the other kernels in terms of validation accuracy and robustness to feature variability. Consequently, the RBF kernel was selected for all experiments and reported results. After hyperparameter optimization, the complete dataset was passed through the CNN feature extractor, and the resulting feature embeddings were used to train the SVM classifier, as illustrated in Figure 1. This two-stage CNN–SVM design leverages the strong representation power of CNNs and the high decision-margin properties of SVMs, resulting in a compact yet effective classification model suitable for real-time FPGA deployment.

### 4.5. Phase 5: Vitis-AI-Based  DPU

To accelerate deep learning tasks, the DPU is specifically designed to execute the computationally intensive operations fundamental to neural networks. A typical DPU comprises multiple parallel processing engines, each optimized for specific neural network operations. These engines work collaboratively to process input data and perform inference, often in real time. In the current work, both the Zynq ZCU102 and the Kria KR260 Kits are utilized for deployment, providing a comparative assessment of performance across different FPGA platforms, occupying approximately 20–27% of LUTs, 17–18% of flip-flops, 28% of BRAM/URAM, and 11–28% of DSP slices, respectively, leaving substantial headroom for additional logic with all timimg constraints, including hold and slack times. The estimated on-chip power is about 8.6 W for the ZCU102 and 4.1 W for the KR260, with junction temperatures around 33–35 °C with sufficient thermal margin.

**Figure 9:**
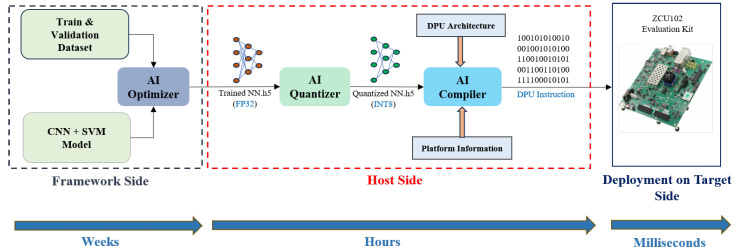
Vitis AI development design flow.

The Vitis-AI-Based DPU is implemented with a host PC (supplied with an Intel (R) Core (TM) i7 2.30 GHz CPU, 16 GB RAM, 1TB hard disk, and Ubuntu 22.04) and a pre-built Vitis-AI docker environment, which uses TensorFlow as the main framework. To generate the necessary highly efficient instruction set and model for the DPU using the Vitis-AI development environment, the process starts with the user DL trained model and proceeds through the Vitis-AI optimizer, quantizer, and compiler [43]. The compiler output encompasses the file of the DPU architecture file containing instructions produced by the Vitis AI compiler. It is important to mention that for each modification in the trained model, the architecture requires the regeneration of the corresponding files [44]. The complete process of the Vitis-AI workflow is shown in Figure 9.

## 5. Results and Discussion

### 5.1. The Proposed Model Results on PC Environment

Before presenting the full experimental results, an ablation study is conducted to evaluate the contribution of each pre-processing stage to the overall system performance. As summarized in Table 5, removing any individual pre-processing component—or omitting the binarization step—consistently reduces validation accuracy by approximately 2–5 percentage points, while inference time remains nearly unaffected. These results demonstrate that the complete proposed pipeline, comprising clutter filtering, squared-absolute transformation, normalization, and binarization, offers the most effective balance between robustness and computational efficiency. The FP32 configuration corresponds to the unquantized CNN–SVM model and is used as the reference baseline for subsequent comparisons with quantized FPGA implementations.

Table 6 compares the proposed CNN–SVM model and the recurrent CNN–RNN variants using the same binarized image inputs. As demonstrated, the proposed CNN–SVM reduces the total parameters from 172,140 to 42,108 relative to the most accurate recurrent baseline (CNN + GRU + SVM), and by up to 86% relative to CNN + Bi-LSTM + SVM, while also achieving the highest training (99.59%) and test (92.79%) accuracies among all models. This confirms that the CNN–SVM hybrid is both more compact and more effective than the considered pipelines on the same binarized dataset.

**Figure 10:**
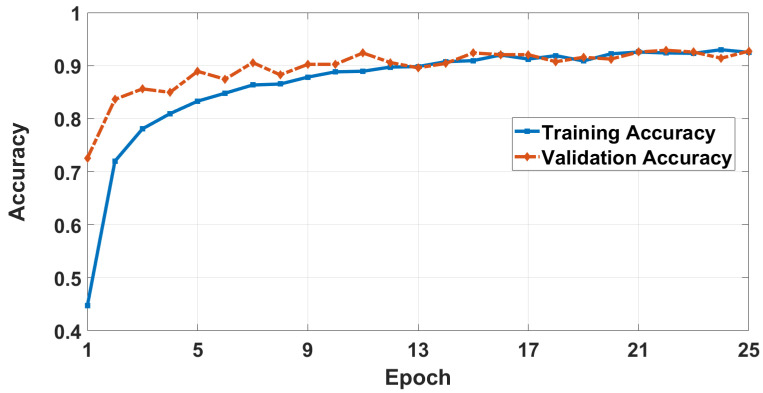
Model accuracy curve in the Vitis AI environment.

**Figure 11:**
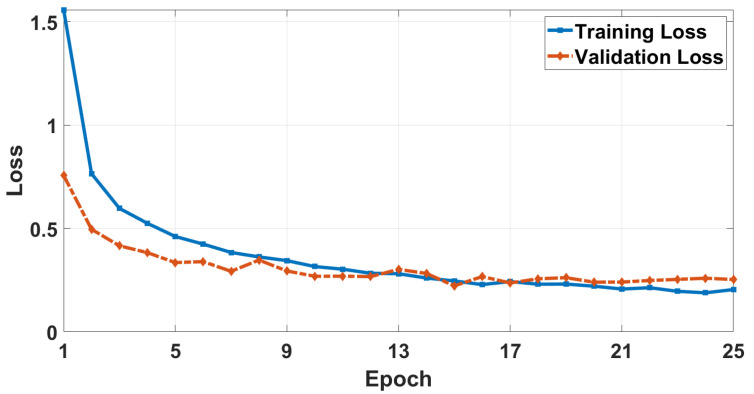
Model loss curve in the Vitis AI environment.

Figure 10 and Figure 11 show the accuracy and loss curves on Vitis-AI environment, respectively. The results indicate that the proposed CNN training model, after 25 epochs, has slightly better results than the CNN-LSTM training model with training and validation accuracies of 93.55% and 93.33%, respectively. However, due to eliminating the LSTM layers, the total number of parameters is reduced to 42,108 instead of 172,140 (reduction percentage of 75.5%). In addition, the SVM training and prediction times, after 100 iterations of the hyperparameter tuning process, are reduced by 29.6% and 32.6%, respectively. This significant improvement enhances the hardware performance when deploying the model as will be shown later.

A more comprehensive comparative analysis is conducted with state-of-the-art models trained using the left radar sensor generated binarized images dataset, as shown in Table 7. Although the CNN–LSTM–ATT model achieves slightly higher validation accuracy and F1-score than the proposed CNN–SVM (97.05% vs. 97.63% accuracy), it relies on a deeper CNN–LSTM–ATT [39] architecture that is significantly more complex and less suitable for efficient FPGA realization. In contrast, the proposed CNN–SVM model is explicitly designed for lightweight deployment, achieving comparable accuracy with far fewer parameters and demonstrating real-time operation on different platforms.

Table 8 presents the execution time for each stage of the proposed framework, including data pre-processing, feature extraction, and classification. Feature extraction is the most computationally intensive stage, consuming 35.29 s, which accounts for approximately 87% of the total processing time. In contrast, data pre-processing for the single range-time data matrix and classification are relatively lightweight, taking 0.487 and 4.8 s, respectively. The results indicate that the majority of computational time is spent on feature extraction, as convolutional operations in CNNs are inherently resource-intensive.

**Figure 12:**
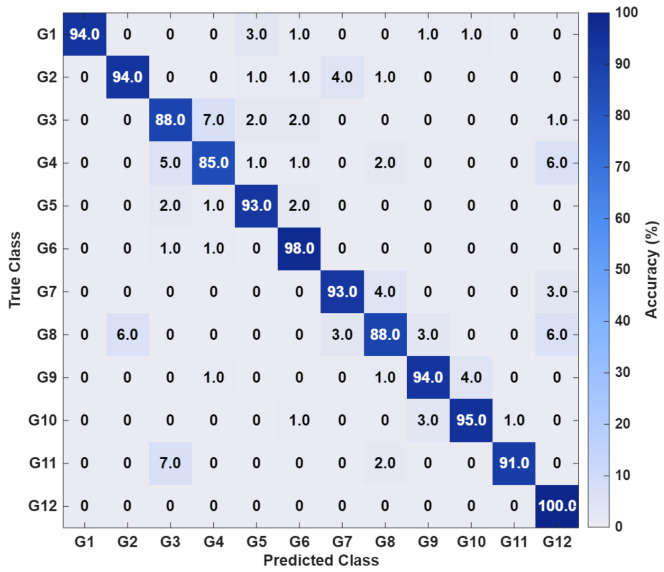
Training confusion matrix results in the Vitis AI Docker environment. Gesture labels correspond to the hand motion classes listed in Table 2.

After host and evaluation board environment setup, the proposed model is trained with the dataset on the Vitis-AI Docker installed environment (Vitis-AI v1.3 and tensorflow v2.10). The confusion matrix of the proposed model (feature extraction + classifier) using the test dataset is shown in Figure 12.

The matrix shows that the best classification accuracy equals to 100% at G12 and the minimum classification accuracy equals to 85% at G4. Notably, over half of the gesture classes achieve accuracy rates exceeding 98%, with an overall precision of 92.13%, recall of 92.10%, and F1-score of 92.06%. The floating-point trained model is quantized using the Vitis-AI quantizer layer and transformed to int8 format to minimize size. The optimum trained model prior to quantization measures 543.3 KB; however, post-quantization, the model measures 224.2 KB. Following quantization, the model is built using the Vitis AI compiler, which produces the necessary files for deployment on the Zynq ZCU102 and the Kria KR260 Kits.

### 5.2. Deployment Results of the Proposed Model on ZCU102 and KR260 Kits

**Figure 13:**
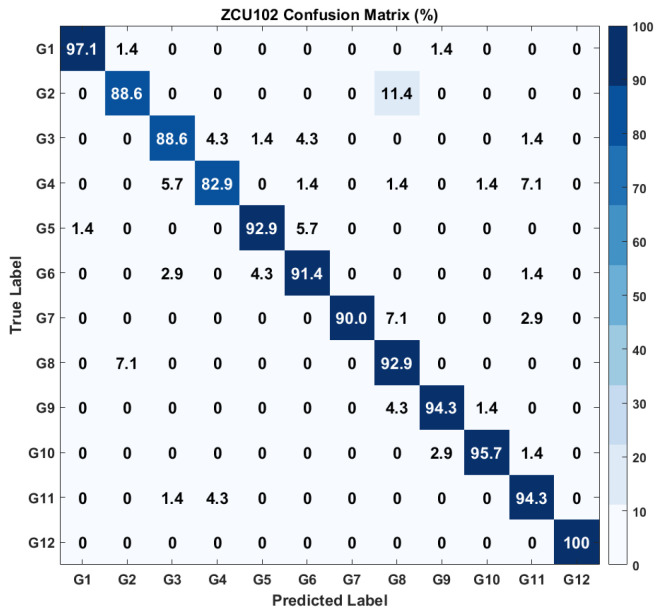
Confusion matrix of the ZCU102 output. Gesture labels correspond to the hand motion classes listed in Table 2.

The results of the model evaluation after deployment on the ZCU102 kit is illustrated through the confusion matrix shown in Figure 13 for the model’s classification performance over 12 distinct classes (G1 to G12) with a total classification accuracy of 92.439%. The majority of classes, such as G1 (97.1%), G9 (94.3%), and G12 (100%), are accurately predicted, demonstrating robust performance. Nonetheless, there exist certain misclassifications, like 11.4% of G2 are erroneously identified as G8 and 7.1% of G8 are projected as G2. Also, classes G4 (82.9%) and G3 (88.6%) exhibit relatively diminished accuracy, indicating the model’s difficulties with these categories. This means that enhancements are necessary to mitigate misclassifications among certain comparable classes for HMR.

**Figure 14:**
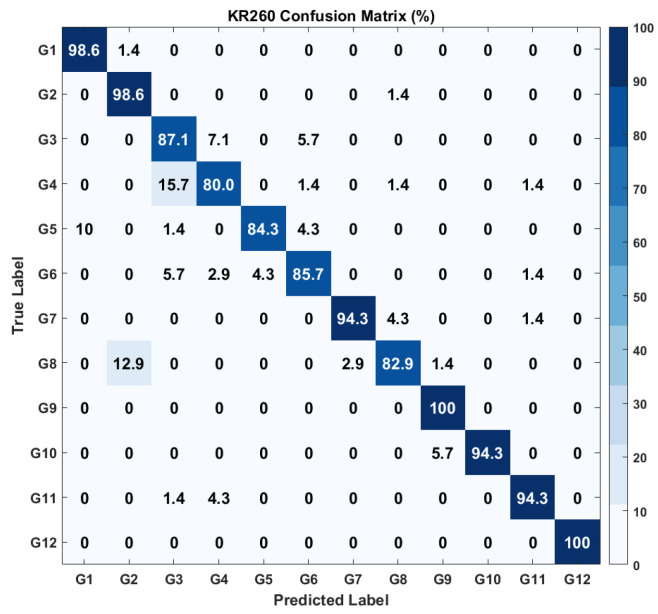
Confusion matrix of the KR260 output. Gesture labels correspond to the hand motion classes listed in Table 2.

On the other hand, the model, after deployment on the KR260 kit, is evaluated through the confusion matrix shown in Figure 14, with a total classification accuracy of 91.67%.

The real-time deployment of the proposed hand motion recognition framework on the Xilinx Kria KR260 platform is demonstrated in the supplementary video available in [48], highlighting the system’s practical applicability and low-latency performance in embedded environments. Similar to results of ZCU102, G1 (98.6%), G9 (100%), and G12 (100%) classes are accurately predicted, demonstrating robust performance. However, there exist certain misclassifications, like 15.7% of G4 are erroneously identified as G3 and 12.9% of G8 are projected as G2. Also, classes G3 (87.1%), G5 (84.3%), and G6 (85.7%) exhibit slightly better accuracy compared to the ZCU102-based model.

**Figure 15:**
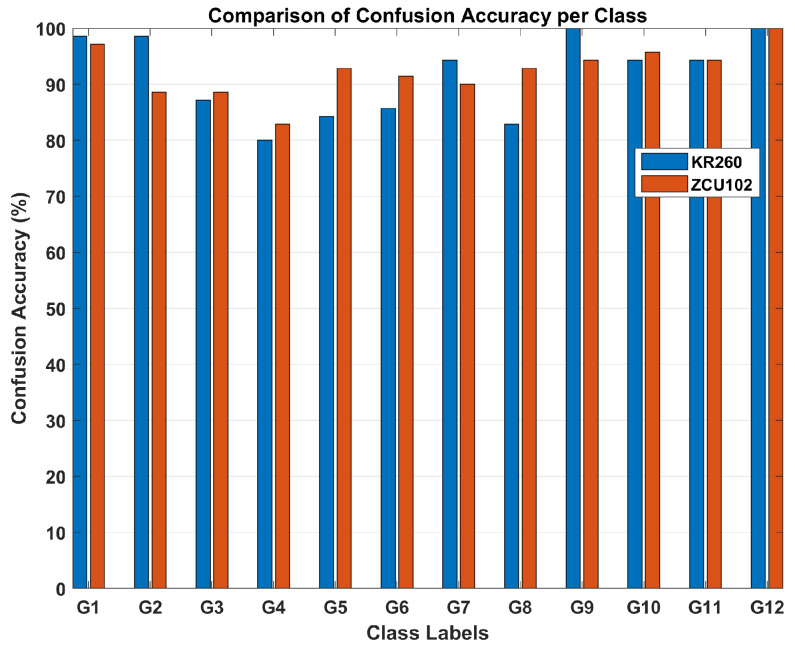
Comparison of classification results for the ZCU102-based model (orange) and the KR260-based model (blue).

Figure 15 presents a comparison highlighting the variations between the models implemented on the ZCU102 and KR260 platforms. For instance, although G2 is accurately identified with a percentage of 98.6% for the KR260-based model, this accuracy is degraded to 88.6% for the ZCU102-based model. In contrast, the classification accuracy of 82.9% for G8 for the KR260-based model is increased to 92.9% for the ZCU102-based model. One contributing factor to the classification confusion between the G2 and G8 classes lies in the similarity of their movement patterns; both involve right-to-left swipes, with G2 characterized by a horizontal movement, while G8 follows a diagonal, down-up diagonal swipe.

To avoid confusion between hardware-level and full-pipeline performance, it is important to clarify that the confusion matrices presented in Figure 13, Figure 14 and Figure 15 correspond solely to the CNN feature extractor deployed on the FPGA after INT8 quantization. These matrices reflect the real-time behavior of the quantized model, which experiences a small expected accuracy drop due to reduced precision. The final classification accuracy of the complete CNN+SVM pipeline is evaluated offline using the reserved test dataset, because the SVM classifier operates on extracted CNN features and does not require hardware execution. The resulting end-to-end test-set accuracies are 96.13% for the ZCU102 platform and 95.42% for the KR260 platform. These values represent the correct final performance of the proposed hybrid model, even though the SVM-based confusion matrices are not included in the manuscript.

**Figure 16:**
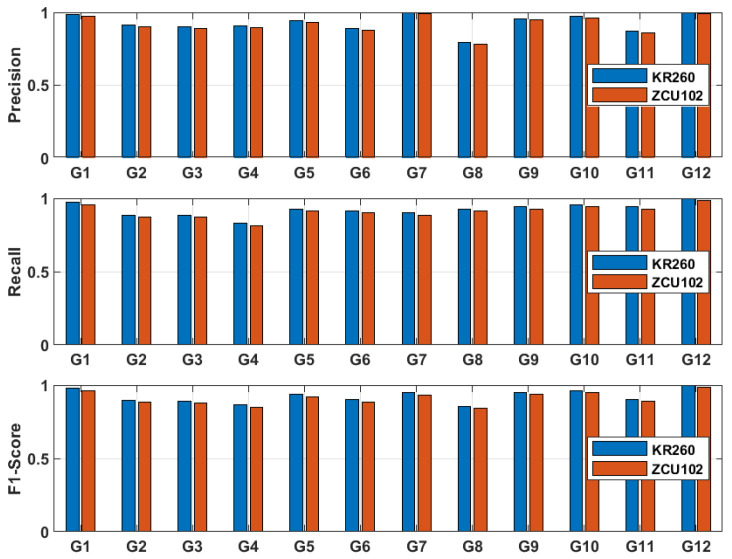
Comparison of different metrics for the ZCU102-based model (orange) and the KR260-based model (blue).

Figure 16 comparison of class-wise precision, recall, and F1-score for the proposed CNN–SVM model on PC and FPGA platforms (ZCU102 and KR260). The metrics reveal that some gestures (G1–G4) maintain high F1-scores across platforms, while a few classes (G5–G8) exhibit slightly lower recall due to residual clutter and gesture similarity.

For deeper investigation, the performance of the proposed model is compared on the two FPGA-based platforms along with the Intel Core i7-12700 PC-based PC platform. The main aspects of the comparison are the classification accuracy, the execution time, and the data throughput, which are listed in Table 9. In terms of classification accuracy, the Intel Core i7-12700 pc platform performs slightly better than the ZCU102-based model and KR260-based model with 1.16% and 1.88%, respectively. This means that, after quantization, less than 2% loss of accuracy has been achieved on both FPGA-based platforms. On the other hand, execution time has decreased with using the ZCU102-based and KR260-based models by 70.3% and 52.8%, respectively. Also, throughput has increased when utilizing the ZCU102-based and KR260-based models by 74.3% and 9.8%, respectively.

It is important to note that although 9.8% throughput increase observed with the KR260-based model may appear modest compared to that achieved using the ZCU102-based model, this difference is intuitively attributed to the number of processing threads, one and four, respectively. Given the cost disparity between the two FPGA kits, the KR260 demonstrates commendable performance, offering a more economically viable solution—particularly considering that its price is approximately one-tenth that of the ZCU102 platform [41,49].

## 6. Conclusions

This paper presents a new framework for radar sensor-based HMR, with an emphasis on addressing challenges such as sensor interference, environmental clutter, and limited dataset diversity. The proposed framework enhances the input quality for deep learning models through a signal pre-processing pipeline involving filtration, normalization, and squared absolute calculation, effectively suppressing clutter before feature extraction. Experimental results demonstrate that the proposed CNN-SVM-based model achieves a high classification accuracy of 98.91%, confirming the effectiveness of the learned features.

The proposed model was deployed on a System-on-Chip (SoC) FPGA using the Vitis AI toolchain, showing less than 2% loss of accuracy on both ZCU102 and KR260 FPGA-based platforms compared to PC-based model. The results show that the hardware implementation yields a 70.3% and 52.8% reduction in execution time, when utilizing ZCU102 and KR260 FPGA-based platforms, respectively. Furthermore, ZCU102 and KR260 FPGA-based platforms achieve 74.3% and 9.8%, respectively, increase in throughput, which underscores the low-latency performance of the models and suitability for real-time applications on resource-constrained platforms. These findings highlight the advantages of the proposed CNN-SVM model over more complex alternatives, particularly on hardware platforms that lack native support for recurrent layers in radar-based HMR. The framework is not only computationally efficient but also scalable and hardware-friendly.

The class-wise confusion matrices and precision–recall–F1 analysis demonstrate that the proposed CNN–SVM framework remains robust under clutter and fixed-point quantization, with only minor degradation on the most ambiguous gestures across FPGA platforms. This behavior is consistent with recent robust recognition approaches in radio, audio, EEG, and LiDAR-based security applications, where careful model design and deployment strategies are essential for maintaining reliability under noise and resource constraints.

Future work will explore the integration of advanced neural topologies such as transformers, improved training methodologies, and optimization techniques, including pruning and quantization-aware training to further enhance model generalization and deployment efficiency in real-world HMR scenarios. In addition, we plan to investigate the fusion of radar data with additional sensors (e.g., inertial measurement units) to improve gesture and motion recognition accuracy.

## Figures and Tables

**Table 1:** Comparison of hand motion recognition technologies.

Feature	Camera-Based [2]	Leap-Motion [5]	Wearable-Sensors [3]	Capacitive-Proximity [6]	Radar-Based [4]
Sensitivity to Lighting	High	High	Low	Low	Low
Privacy Concerns	High	Medium	Low	Low	Low
Real-Time Processing	Medium	High	Medium	Medium	High
Ease of Use	Medium	High	Low	Medium	High
Cost	Low	Medium	High	Medium	Medium

**Table 2:** Hand motion Codes and Descriptions.

Code	Gesture Class
G1	Left-Right swipe (LR-swipe)
G2	Right-Left swipe (RL-swipe)
G3	Up-Down swipe (UD-swipe)
G4	Down-Up swipe (DU-swipe)
G5	Left-Right-Up-Down diagonal swipe (diag-LR-UD-swipe)
G6	Left-Right-Down-Up diagonal swipe (diag-LR-DU-swipe)
G7	Right-Left-Up-Down diagonal swipe (diag-RL-UD-swipe)
G8	Right-Left-Down-Up diagonal swipe (diag-RL-DU-swipe)
G9	Clockwise rotation (CW-rotation)
G10	Counterclockwise rotation (CCW-rotation)
G11	Inward push
G12	Empty gesture

**Table 3:** Architecture sensitivity analysis for CNN depth and regularization settings.

Configuration	Accuracy (%)	Params	FPGA Utilization
2 conv layers (16–32 filters)	92.8	0.24 M	Low
3 conv layers (16–32–64)	96.1	0.41 M	Moderate
4 conv layers (16–32–64–128)	97.4	1.09 M	High
3 conv layers, kernel 3 × 3	95.4	0.37 M	Moderate
3 conv layers, dropout 0.1	94.7	0.41 M	Moderate
3 conv layers, dropout 0.5	93.8	0.41 M	Moderate

**Table 4:** Detailed structure of the proposed CNN model.

Layer	Output Shape	Details
Input	75 × 75 × 1	-
CNN_1		
Conv2D	38 × 38 × 16	Filter Size: 4 × 4, Filters: 16; Stride: 2 × 2; Padding: Same
MaxPooling2D	19 × 19 × 16	Pool Size: 2 × 2
Dropout	19 × 19 × 16	Rate: 0.2
CNN_2		
Conv2D	10 × 10 × 32	Filter Size: 4 × 4, Filters: 32; Stride: 2 × 2; Padding: Same
MaxPooling2D	5 × 5 × 32	Pool Size: 2 × 2
Dropout	5 × 5 × 32	Rate: 0.2
CNN_3		
Conv2D	3 × 3 × 64	Filter Size: 4 × 4, Filters: 64; Stride: 2 × 2; Padding: Same
MaxPooling2D	1 × 1 × 64	Pool Size: 2 × 2
Dropout	1 × 1 × 64	Rate: 0.2
Flatten	64 × 1	-
Dense	12 × 1	Activation: Softmax

**Table 5:** Ablation results for pre-processing and quantization steps.

Variant	Validation Accuracy (%)	Inference Time (ms)
Full pipeline (proposed)	93.33	0.45
No filtering	90.12	0.44
No squared-absolute	91.28	0.44
No normalization	88.77	0.43
No binarization (grayscale images)	90.45	0.46
Without quantization (FP32)	94.82	0.45

**Table 6:** Comparison of trained models using the generated binarized dataset.

Model	Total Params	Total FLOPs/Sample	Train Acc.	Test Acc.
CNN + LSTM + SVM [28]	172,140	3,227,952	98.63%	91.27%
CNN + Bi-LSTM + SVM [45]	302,000	3,484,752	98.40%	91.87%
CNN + GRU + SVM [46]	138,000	3,163,752	98.88%	92.20%
CNN + RNN + SVM [47]	115,000	3,035,352	98.81%	91.47%
CNN + SVM (Proposed)	42,108	2,967,552	99.59%	92.79%

**Table 7:** Benchmarking performance of hand motion recognition models trained on the Left-Radar dataset.

Model	Train Accuracy (%)	Validation Accuracy (%)	Precision (%)	Recall (%)	F1-Score (%)
CNN-ETs [37]	88.86	81.52	82.14	81.52	81.68
CNN-LSTM-SVM [28]	96.30	93.09	93.24	93.10	93.44
CNN-Softmax [38]	98.33	91.98	91.67	91.52	91.45
GPNAS [29]	–	96.50	–	–	–
CNN-LSTM-ATT [39]	–	97.05	97.05	97.04	97.02
Proposed Model	98.91	97.63	92.13	92.10	92.06

**Table 8:** Time cost of each processing stage.

Processing Stage	Execution Time (s)
Data Pre-processing	0.487
Feature Extraction	35.29
Classification	4.80
Total Processing Time	40.577

**Table 9:** Comparative analysis of the quantized CNN feature extractor on different hardware platforms.

Metric	Intel i7-12700	ZCU102	KR260
Average Accuracy (%)	93.55	92.39	91.67
Execution Time (ms)	620	184.2	292.5
Throughput (images/s)	2614.67	4559.04	2871.82
Number of Threads	20	4	1

## Data Availability

The data and code supporting the reported results are available at https://drive.google.com/drive/folders/1UKPcUlqxoRDC60PSAAISp6-CpCramSIJ?usp=sharing (accessed on 16 December 2025).

## Abbreviations

The following abbreviations are used in this manuscript:

HMR	Hand Motion Recognition
TFR	Time–Frequency Representation
AI	Artificial Intelligence
DL	Deep Learning
IR	Impulse Radar
UWB	Ultra-Wideband
RoI	Region-of-Interest
PRIs	Pulse Repetition Interval
LSTM	Long Short-Term Memory
CNN	Convolutional Neural Network
SVM	Support Vector Machine
FPGA	Field-Programmable Gate Array
MPSoc	Multi-Processor System on Chip
DPU	Deep-learning Processor Unit
